# Stopping to food can reduce intake. Effects of stimulus-specificity and individual differences in dietary restraint^☆^

**DOI:** 10.1016/j.appet.2014.11.006

**Published:** 2015-02-01

**Authors:** Natalia S. Lawrence, Frederick Verbruggen, Sinead Morrison, Rachel C. Adams, Christopher D. Chambers

**Affiliations:** a School of Psychology, College of Life and Environmental Sciences, University of Exeter, Exeter EX4 4QG, UK; b School of Psychology, Cardiff University, Park Place, Cardiff CF10 3AT, UK

**Keywords:** Response inhibition, Cognitive training, Stop Signal task, Overeating, Food intake, Dietary restraint

## Abstract

Overeating in our food-rich environment is a key contributor to obesity. Computerised response-inhibition training could improve self-control in individuals who overeat. Evidence suggests that training people to inhibit motor responses to specific food pictures can reduce the subsequent choice and consumption of those foods. Here we undertook three experiments using the stop-signal task to examine the effects of food and non-food related stop-training on immediate snack food consumption. The experiments examined whether training effects were stimulus-specific, whether they were influenced by the comparator (control) group, and whether they were moderated by individual differences in dietary restraint. Experiment 1 revealed lower intake of one food following stop- vs. double- (two key-presses) response training to food pictures. Experiment 2 offered two foods, one of which was not associated with stopping, to enable within- and between-subjects comparisons of intake. A second control condition required participants to ignore signals and respond with one key-press to all pictures. There was no overall effect of training on intake in Experiment 2, but there was a marginally significant moderation by dietary restraint: Restrained eaters ate significantly less signal-food following stop- relative to double-response training. Experiment 3 revealed that stop- vs. double-response training to non-food pictures had no effect on food intake. Taken together with previous findings, these results suggest some stimulus-specific effects of stop-training on food intake that may be moderated by individual differences in dietary restraint.

## Highlights

• We examine whether cognitive training (response inhibition) modifies food intake.
• Food stimulus-specific training can influence food intake.
• These effects are more pronounced in restrained eaters.
• General training to non-food stimuli did not influence food intake.

## Introduction

We are in the midst of an obesity epidemic. Rates of obesity (BMI ≥ 30 kg/m^2^) in adults have increased three- to four-fold in the last 30 years, rising from 6% (UK) and 15% (US) in 1980 to 26% and 35% respectively today, with most adults (60–70%) now overweight or obese (Department of Health, 2012; Flegal, 2005; Flegal, Carroll, Kit, & Ogden, 2012). Over-eating in the context of an increasingly food-rich environment is a key contributor to rising obesity levels (Hill, Wyatt, Reed, & Peters, 2003), with large individual differences in susceptibility to our shared ‘obesogenic’ environment (Carnell, Kim, & Pryor, 2012; Grucza et al., 2010).

We and others have recently shown that individual differences in response to food pictures in reward/motivation-related brain regions are positively associated with food intake (Lawrence, Hinton, Parkinson, & Lawrence, 2012) and can predict weight gain in healthy and obese individuals (Demos, Heatherton, & Kelley, 2012; Murdaugh, Cox, Cook, & Weller, 2012). Importantly, however, individual differences in self-control can moderate the impact of heightened food cue-reactivity on weight over the longer-term: Individuals who show a strong reward-related response to foods combined with low levels of self-control are particularly susceptible to gaining weight, whereas those with effective self-control appear to be protected (Lawrence et al., 2012; Nederkoorn, Houben, Hofmann, Roefs, & Jansen, 2010). These findings are consistent with evidence linking impulsivity to obesity in adults and children (e.g. Nederkoorn, Braet, Van Eijs, Tanghe, & Jansen, 2006a; Nederkoorn, Smulders, Havermans, Roefs, & Jansen, 2006b). In particular, poor motor response inhibition, measured using stop-signal and go/no-go tasks (Verbruggen & Logan, 2008a), is associated with increased BMI (Nederkoorn et al., 2006a, 2006b) and increased food intake in the lab (Guerrieri et al., 2007). Furthermore, the inhibition of responses to appetising food pictures may be particularly compromised in overweight individuals (Batterink, Yokum, & Stice, 2010; Houben, Nederkoorn, & Jansen, 2014; Nederkoorn, Coelho, Guerrieri, Houben, & Jansen, 2012).

These findings have prompted studies examining whether inhibitory control can be strengthened through training in order to influence people's eating behaviour. Several studies have now demonstrated that one session of inhibiting simple motor responses to pictures of snack foods, in the context of go/no-go or stop-signal tasks, can reduce subsequent consumption or choice of those foods (Houben, 2011; Houben & Jansen, 2011; Van Koningsbruggen, Veling, Stroebe, & Aarts, 2014; Veling, Aarts, & Papies, 2011; Veling, Aarts, & Stroebe, 2013a, 2013b). Similar effects have been demonstrated for alcohol consumption following one session of inhibiting responses to pictures of alcohol (Bowley et al., 2013; Houben, Havermans, Nederkoorn, & Jansen, 2012a; Houben & Jansen, 2011; Houben, Nederkoorn, Wiers, & Jansen, 2011; Jones & Field, 2013). Although the precise methods and results of these studies differ (e.g. some show only immediate effects whilst others demonstrate longer-lasting reduction of intake), they suggest that response-inhibition training has the potential to help reduce excessive or impulsive eating and drinking behaviour.

Several questions about these training effects remain, including their mechanism of action, cue-specificity, duration, influence of participants' awareness of the training, and the moderation of effects by individual differences. For example, in the three published studies on food intake, the effects of response-inhibition training were moderated by individual differences in inhibitory control ability (Houben, 2011) or dietary restraint (Houben & Jansen, 2011; Veling et al., 2011), with stronger training effects observed in more impulsive or restrained individuals. Impulsivity and dietary restraint are themselves risk factors for overeating and overweight (Johnson, Pratt, & Wardle, 2012; Nederkoorn et al., 2006b), suggesting that response-inhibition training to food pictures may specifically help to reduce overeating in vulnerable individuals, which supports its therapeutic potential.

In terms of underlying mechanisms, it is important to clarify whether response-inhibition training effects are mediated through a general strengthening or priming of inhibitory control, consistent with evidence for ‘inhibitory spillover’ between psychological or behavioural domains (Berkman, Burklund, & Lieberman, 2009; Tuk, Trampe, & Warlop, 2011), or whether the effects are specific to motivationally-salient stimuli. For example, if equivalent reductions in food intake could be achieved through general response-inhibition training using non-food stimuli, this would be advantageous as it would avoid exposing at-risk individuals to high-incentive food cues, which alone can increase food consumption (Fedoroff, Polivy, & Herman, 1997; Lawrence et al., 2012). Preliminary findings suggest, however, that such general response-inhibition training is ineffective in reducing the immediate consumption of food (Guerrieri, Nederkoorn, & Jansen, 2012) or alcohol (Jones & Field, 2013), indicating that training effects may be stimulus-specific.

Stimulus-specific effects of response-inhibition training have also been demonstrated by comparing the relative intake of foods associated with going or stopping in a repeated-measures design (Houben, 2011). Recent investigations into the mechanism underlying response-inhibition training also support stimulus-specific effects: Affective cues associated with no-go responses show a reduction in rated valence (Doallo et al., 2012; Veling et al., 2013a; Veling, Holland, & Van Knippenberg, 2008) and more negative implicit affective reactions (Houben, Nederkoorn, & Jansen, 2012b; Veling & Aarts, 2009). In addition, the automatic motor impulses activated by cues are modified through response-inhibition training (Verbruggen & Logan, 2008a), although it is unclear whether this specific mechanism also influences food consumption (Houben et al., 2012a; Veling et al., 2011). Thus, further research is needed to clarify the stimulus-specificity and mechanisms underlying the effects of response-inhibition training in order to optimise this behavioural intervention prior to testing it in clinical or real-world contexts.

Here we report three experiments, which progressively build upon one another, to examine the effects of the stimulus-specificity of response-inhibition training on immediate snack food consumption, along with the influence of the comparator (control) condition and individual differences in dietary restraint. Dietary restraint can be defined as the tendency to deliberately restrict food intake with the aim of losing weight or preventing weight gain; however, this is often unsuccessful and restrained eaters typically eat and weigh more than unrestrained eaters (see Johnson et al., 2012 for a recent review). Whilst different measures of restraint have been used in prior studies, findings agree that restrained eaters show stronger effects of food response-inhibition training in reducing food intake (Houben & Jansen, 2011; Veling et al., 2011) so in all three experiments we examined whether individual differences in restraint moderated the effects of training on food intake. We also used a funnelled debriefing interview to gauge participants' awareness of the stop-associations in the training tasks.

In Experiment 1, we adopted a simple between-subjects design to examine the effect of stop- vs. double-response training to food stimuli on subsequent crisp consumption. In the stop condition, participants performed a variant of the stop-signal reaction time task (Logan, 1994; Verbruggen & Logan, 2008b). In this task, participants have to withhold their response to a go stimulus when an extra signal is presented. The double-response control condition required participants to carry out the normal response followed by an additional response when an extra signal was presented (see methods), which has been used a control condition for stop-signal training in our previous studies (Verbruggen, Adams, & Chambers, 2012). The double-response task controls for the additional attentional and action updating components associated with the stop-signal training task, but in a way that does not require outright response inhibition (Dodds, Morein-Zamir, & Robbins, 2011; Verbruggen, Aron, Stevens, & Chambers, 2010). Standard control conditions do not do this; they either contain all ‘go’ trials (with no signals being presented at all) or they require random response inhibition (i.e. all stimuli are randomly associated with stop and go signals). In Experiment 2, we included a third control condition. In this condition, participants were instructed to ignore additional signals, and execute a single response on each trial. In a taste test, participants were given two foods to eat (crisps and chocolate), only one of which was associated with stop-, double-response, or ignore signals, to enable both between- and within-subjects comparisons of intake. In Experiment 3, we examined the effects of stop- vs. double-response training to non-food stimuli on subsequent consumption of the same two foods as in Experiment 2.

## Experiment 1 – stop- vs. double-response training effects on consumption of one food

The first study used a modified stop-signal task (SST) to train participants to inhibit or make double-responses to images of foods, in particular to one subsequently presented food (crisps). We predicted that consistent stimulus-stop associations would affect participants' consumption when they were presented with crisps in an ad-libitum snacking phase.

### Methods and materials

#### Participants

Sixty-five participants (39 women) were recruited from the student and staff population at Cardiff University, using online advertising and the Psychology department's online experimental management system. Participants were aged 18–46 years. Participants were semi-randomly assigned to groups keeping age, gender, and time-of-day seen balanced between groups. All procedures in this and subsequent experiments were approved by the Cardiff University School of Psychology Research Ethics Committee, and all participants signed a written statement of informed consent.

#### Apparatus

The experiment was conducted using a Pentium 3 PC running Matlab software (MathWorks , 2011). Stimuli were presented on a 17-inch monitor and responses were collected via a keyboard. The stimuli were simple pictures of food and non-food objects (presented at a visual angle of 13.47) presented on a white background. Some of the pictures had previously been used in fMRI studies of food cue-reactivity, and the food pictures had been rated as pleasant (Beaver et al., 2006; Lawrence et al., 2012). These were supplemented by similar, additional stimuli selected from the Internet to ensure sufficient exemplars in each food and non-food category (see below for details). Food and non-food images were matched as closely as possible for size, colour and visual complexity.

#### Task

**Fig. 1:**
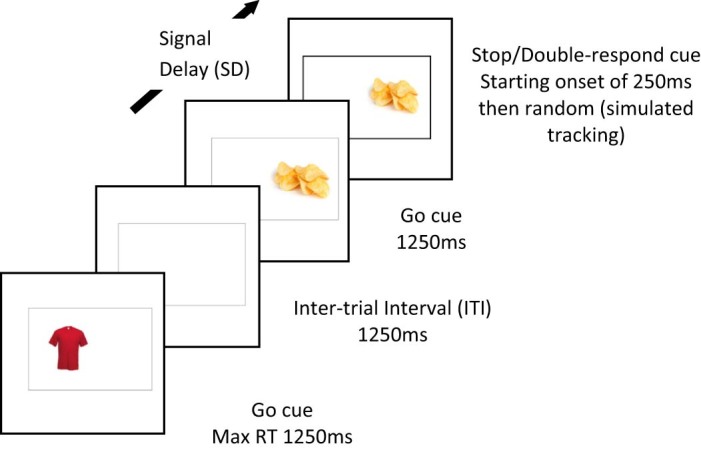
Schematic of the food-associated stop-/double-response task. Participants pressed a key to indicate whether the object appeared to the left or right of the centre of the screen. If the frame surrounding the object became bold after a variable delay, they had to withhold their response (stop group) or execute an additional response (double-response group).

Pictures were presented within a black rectangular frame in the centre of the screen. Pictures were presented to the left or right of the centre of the screen within the rectangle (see Fig. 1). For standard go trials participants were instructed to press left/right response keys (“J” and “K” on the keyboard, respectively) with the right index and middle finger, respectively. On signal trials the lines of the rectangle became bold (see Fig. 1) and participants were instructed to either withhold their response for that picture (stop-group) or carry out their normal left/right response followed by an additional response (pressing the space bar with the right thumb; double-response group, Verbruggen et al., 2012). The double-response task therefore controlled for stimulus exposure and for foods being associated with signal trials, which we presumed would increase the attentional salience of food pictures. Each trial started with one picture being presented within the rectangular frame for 1250 ms, followed by a 1250 ms inter-trial interval consisting of the rectangular frame only. On go or double-response trials, participants were instructed to respond to the picture location within the 1250 ms picture presentation. The experiment consisted of ten blocks of 48 trials.^1^ Participants were observed during the first block to check that they were following instructions. There was a 30 second break every two blocks.

**Fig. 2:**
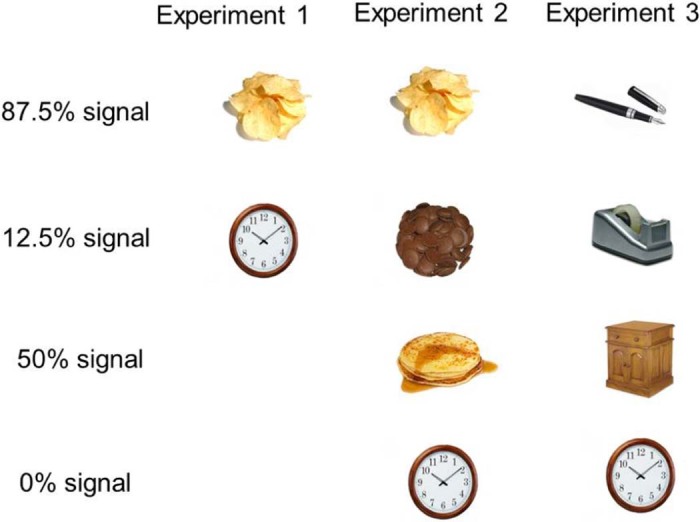
Stimulus–signal associations in each experiment. Different categories of pictures were associated with signal trials (a bold frame around the image) using the probabilities shown. Signal trials required either a stop- or double-response, or a standard go response (‘ignore-control’ group in Experiment 2). In Experiment 1, half of the food items were crisps. In Experiment 2 and 3, the categories associated with signal trials 87.5% or 12.5% of the time were counterbalanced across subjects.

Pictures from the following categories were presented in a random order: Food (including 8 exemplars of crisps and 8 exemplars of other foods; pasta, pizza, cakes, pancakes), or non-food items (32 exemplars; household/garden objects and clothes e.g. clock, rake, gloves). Each exemplar (48 in total) was presented once per block. A stop/double signal (bold frame) was presented on 33% of trials in each block (160/480 signal trials overall). The majority of signals (140 trials; 87.5%) occurred during the presentation of food (70 signals occurred on crisp trials, and 70 signals occurred on the other food trials) pictures, with the remaining signals (20 trials; 12.5%) occurring during the presentation of non-food pictures. This weighting of signals to stimulus categories was designed to encourage associative learning (i.e. food-inhibition associations) whilst maintaining task difficulty and attention. We introduced a small number of catch trials (i.e. food images to which they had to respond) because we wanted to reduce participants' explicit awareness of the association, and encourage the formation of specific stimulus–response associations rather than new rules (for a discussion of the distinction between stimulus–response and propositional learning, see e.g. McLaren et al., 2013). Most non-food pictures were therefore standard go trials (one key-press), whilst food pictures were associated with a stop/double signal on 87.5% of trials. Figure 2 illustrates the associations between signals and specific picture categories in all three experiments.

The stop/double signal delay (SSD) was initially set at 250 ms and continuously adjusted according to a ‘simulated’ tracking procedure based on the last no-signal (go) trial reaction time (see also Verbruggen & Logan, 2009a). This ‘simulated’ tracking procedure was as follows, where X is the reaction time of the last no-signal trial: If X > SSD + 200, then SSD = SSD + 25 (viz. signal-inhibit trial) else SSD = SSD − 25 (viz. signal-respond trial). We used this simulated tracking procedure in case subjects became aware of the signal–stimulus associations. The catch trials and simulated tracking procedure ensured that the task remained a stop-signal task, placing demands on ‘action cancellation’ (inhibition of an initiated response) rather than it becoming a qualitatively different ‘action restraint’ task, typically elicited in go/no-go tasks (Schachar et al., 2007).

#### Procedure

Participants were informed that this was a study of individual differences in motivation and reward that involved completing computerised tests of attention and reaction time, along with questionnaires about personality and mood. They were seen between 1pm and 6pm and instructed to refrain from eating for three hours before the study because the cognitive processes under investigation “are sensitive to blood glucose levels and we want all participants to have similar levels of glucose deprivation”. Whilst studies into ego-depletion indicate that this may be true (Masicampo & Baumeister, 2008, but see Inzlicht, Schmeichel, & Macrae, 2014), these instructions were designed to limit participants' awareness that we were really looking at hunger state and food consumption.

Upon arrival, participants gave written informed consent, and completed two state measures. First they rated their current feelings of hunger, fullness and desire to eat using 100 mm visual analogue scales (Flint, Raben, Blundell, & Astrup, 2000; Yeomans, 2000), followed by the positive and negative affective schedule to measure current mood state (PANAS; Watson, Clark, & Tellegen, 1988). Participants then performed the stop-signal task, which lasted approximately 20 minutes. Following the task, participants were presented with a pack of filler questionnaires measuring mood and personality traits (unrelated to food/eating) along with some refreshments; a large, clear plastic bowl filled with 125 g of crisps (as in Lawrence et al., 2012) and a glass of water. The crisps were Tesco Ready Salted Crisps, 5.45 kcal/g. After 15 minutes any completed questionnaires were collected, the food was removed, and participants were asked to complete the final eating-related questionnaires including the Dutch Eating Behaviour Questionnaire (DEBQ), a 33 item questionnaire that measures emotional, restrained and external eating behaviour (Van Strien, Frijters, Bergers, & Defares, 1986) and the Food Craving Trait Scale (FCT), a 21 item scale to measure the extent to which participants generally crave food (Nijs, Franken, & Muris, 2007). The DEBQ restrained eating score was used in moderated regression analyses (see below). The crisps were removed and weighed in another room.

At the end of the experiment all remaining questionnaires were collected and participants were asked a series of questions via a funnelled debriefing interview to gauge study awareness. Specific questions asked participants; (i) whether they had noticed anything in particular in the computer task, (ii) whether they had noticed anything about when they had to stop (or make a double response) and if not, then (iii) whether they thought the stop (or double) signals were distributed evenly (and if not evenly, which pictures were associated with signals). Participants were then asked whether they thought the task had influenced their questionnaire responses or snacking behaviour and if so, how. Finally, participants were asked whether they had previously participated in similar studies where they had been required to inhibit responses to pictures of food or were offered food to eat – data from these participants were subsequently excluded. At the end of the session, participants' height and weight was measured in order to calculate their body mass index (BMI; kg/m^2^), and they were debriefed and paid £6.

### Statistical analyses

In all three studies, participants were excluded if they met one of the following criteria: Reported having previously participated in a similar study; had eaten within three hours of starting the experiment; were unable to complete the taste test (e.g. due to fasting); had incomplete task performance data; or were outliers (more than 3 standard deviations from their group mean) in terms of food intake^2^ (total kcal) or task performance (go or signal trials). Performance outliers for signal trials were identified using a threshold of lower than 3 SDs from the group mean in the double or control groups; however, a more conservative threshold of 2 SDs from the group mean was applied to participants in the stop groups. This was because 3 SDs from the mean included 0% stop-signal accuracy in some studies, and we wanted to ensure that only participants who showed some evidence of successful stopping (e.g. equivalent to at least 55.4% in Experiment 1) were included in the analysis. We believe that it is only possible to examine the effects of stop-training on eating behaviour in participants who demonstrate some engagement with the training task, i.e. some successful stopping (see Verbruggen & Logan, 2008a). These exclusions (n = 11; details provided in Supplementary Table S4) resulted in a final sample of 54 in Experiment 1 (n = 29 in the inhibition condition, n = 25 in the double response condition). This final sample size yielded 80% power to detect pairwise group differences of Cohen's d ≥ .78.

Continuous variables (age, BMI, dietary restraint, calories consumed) were compared using between-groups ANOVA, with α = .05. Categorical variables (gender) were compared using chi-squared tests. A moderated regression was also conducted to examine relationships between training condition (coded as a dummy variable), dietary restraint (DEBQ) and calorie intake. The modprobe SPSS macro (Hayes & Matthes, 2009) for exploration of interactions in multiple regression was used, with training condition (stop or double, dummy-coded) as the focal predictor variable, dietary restraint as the moderator variable, and calorie intake as the dependent variable.

All data files are deposited in the University of Exeter's Open Research Exeter repository under the following identifier: (http://hdl.handle.net/10871/15856).

### Results

The inhibition and double response groups were well matched for age (*M* = 24, *SD* = 5.2), sex (59% female), BMI (*M* = 22.9, *SD* = 3.8; range 17–36.2), dietary restraint (*M* = 2.54, *SD* = 0.92), trait food craving (*M* = 59.54, *SD* = 17.47), hours since last food consumption (*M* = 4.74, *SD* = 3.2) and state measures (hunger and mood) (all ps > .1). Full descriptive information and significance tests between groups are provided in Supplementary Table S1.

Performance on standard go trials was similarly high in both groups (Supplementary Table S1) but mean RT on correct go trials was faster in the double-response than stop group (*M* = 424.9, *SD* = 74.9 vs. *M* = 599.5 *SD* = 150.9, respectively), suggesting response strategy adjustments in the stop context (e.g. Aron, 2011; Verbruggen & Logan, 2009a). Accuracy on signal (double or stop) trials was higher in the double-response than stop group (*M* = 95.8, *SD* = 3.2 vs. *M* = 78.6 *SD* = 10.4). Because the double-response could be executed during the whole response interval, it is not surprising that accuracy on signal trials was higher in the double-response than in the stop condition. All performance data and statistics are included in Supplementary Table S1.

**Fig. 3:**
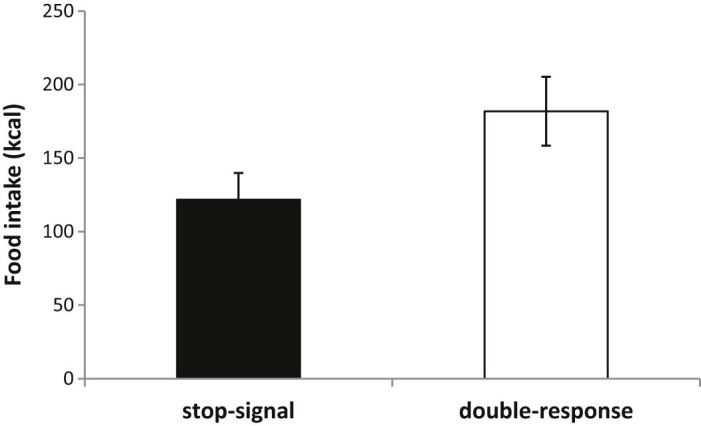
Crisp consumption in participants performing a food-related stop-signal task (Experiment 1) relative to those performing a food-related double-response task. Graphs display group mean intake ± standard errors.

The main purpose of this experiment was to examine stop-training effects on crisp consumption. Participants in the stop-group consumed significantly fewer calories than participants in the double-response group (mean difference: −60.1 kcal, 95% confidence interval (CI) = −1.51 to −118.68 kcal; F(1, 53) = 4.24, p = 0.045, η^2^p = 0.075, Cohen's d = 0.56; see Fig. 3). Thus stop- relative to double-response training was associated with 33% less ad-libitum crisp consumption. Dietary restraint did not interact significantly with the effect of training in the moderated regression analysis (t = −.16, p = .88; Δ R^2^ = .0004).^3^ Therefore in this experiment, there was a main effect of training condition on calorie intake but this effect was not significantly moderated by individual differences in dietary restraint.

We examined whether inhibition accuracy to food in the stop-group improved over time and whether this was associated with ad-lib food consumption (Jones & Field, 2013). There was an increase in the proportion of successful food-stop trials from early (first two blocks; *M* = 0.65, *SD* = 0.22) to late (last two) blocks (*M* = 0.91, *SD* = 0.1; t(28) = 6.52 p < .001), consistent with learning the food-stop associations. However, there was no significant correlation between ad-lib consumption and either overall food-stop accuracy (r(29) = .16, p = .42) or the improvement in food-stop accuracy from early to late blocks (r(29) = .35, p = .065).

### Awareness of stimulus-specificity of training task

During the funnelled debriefing procedure at the end, the majority of participants (83%) reported noticing that signals were associated with pictures of food. Due to the small number of participants (17%) reporting no awareness of signal-food associations, we did not analyse how this influenced performance or food consumption. The proportion of “aware” participants was similar in both groups (Supplementary Table S1). Importantly, no participants guessed that the aim of the study was to examine the effect of stop-training on reducing subsequent food consumption. When asked directly, the majority of participants (61%) did not think that the task influenced how much they snacked afterwards, whereas 39% thought that the food images in the task made them feel hungrier and may have made them eat more. This distribution of responses did not differ significantly between groups (Supplementary Table S1).

### Discussion

Experiment 1 demonstrated that participants who were trained to predominantly inhibit motor responses to pictures of food ate significantly less crisps than participants who were trained to execute a double-response to food pictures. This replicates previous reports of reduced food intake following one session of food-associated stop or no-go vs. go (or inconsistent go/no-go) training (Houben, 2011; Houben & Jansen, 2011; Veling et al., 2011). Previous studies have demonstrated training effects on intake in certain individuals, namely those high in dietary restraint (Houben & Jansen, 2011; Veling et al., 2011) or low in inhibitory control (Houben, 2011). In contrast, we found no significant interaction between training effects and dietary restraint in this study; instead a main effect of training in an unselected sample was observed. This could be due to the training of a general association between food (as a category) and inhibition in this study, in contrast to more stimulus-specific associations in some earlier studies (Houben, 2011; Houben & Jansen, 2011). There were also differences in the consumption test between this and earlier studies; we measured voluntary ad-libitum intake of one food (crisps, as in Lawrence et al., 2012) whereas others measured intake of a variety of food in a bogus taste test (Houben, 2011; Houben & Jansen, 2011) or voluntary consumption of one food outside of the lab over a 24-hour period (Veling et al., 2011).

It is not clear whether the main effect of training in this experiment was due to reduced consumption in the stop-group or increased consumption in the double-response group. Evidence suggests that general (non food-related) ‘impulsivity training’ (successive increases in go responding) can increase food consumption in a subsequent taste test relative to a neutral reading condition (Guerrieri et al., 2012). Moreover, a recent study demonstrated increased food choice and positive evaluation following food cue-approach training (pairing specific foods with auditory ‘go’ cues; Schonberg et al., 2014). Furthermore, go- relative to no-go training to alcohol caused a near-significant increase in a psychophysiological index of approach motivation (frontal EEG asymmetry) to alcohol pictures (Bowley et al., 2013). These studies suggest that responding to food pictures may increase subsequent food consumption through general and food-specific motor disinhibition, and increased positive evaluation and approach motivation for ‘go’ foods. It is possible, therefore, that our food double-response training had similar disinhibition effects on food intake, by combining ‘impulsivity’ and food-related approach training. The faster go RTs in our double-response relative to stop-group are consistent with the effects of impulsivity training (Guerrieri et al., 2012; Guerrieri, Nederkoorn, Schrooten, Martijn, & Jansen, 2009).

In our next experiment we therefore included a second control condition, in which participants were instructed to simply perform the primary location identification task and ‘ignore’ the bold frame signal. We attempted to control for attention and stimulus salience across tasks by continuing to present signals using the same associations with images in the ‘ignore’ task as in the stop- and double-response tasks. The ‘ignore’ condition was therefore a single-response go condition, but we refer to it as ‘ignore’ for consistency with our previous work (Verbruggen et al., 2012) and to make it clear that it involved signals additional to the main stimulus that were ignored by the participant. The ignore condition was intended to provide a baseline for establishing whether stop training reduced consumption relative to food cue exposure with single responses, or whether double-response training increased it.

We also wanted to further investigate the stimulus-specificity of response-inhibition training and so adopted a mixed between- and within-subjects design. Experiment 2 measured the consumption of two foods, only one of which was associated with signal trials. Finally, to ensure that all participants ate some of the foods and to provide a stronger justification for offering food in the experiment, the foods were presented as part of a bogus taste test, using the same questions as those used in previous studies (Houben, 2011; Houben & Jansen, 2011).

## Experiment 2 – stop-, double-response and ‘ignore’ training effects on consumption of two foods

A similar modified stop-signal task was used to train participants to stop (or make double-responses) to images of one of two subsequently presented foods (crisps or chocolate, counterbalanced); the other food was associated with standard go responses. We predicted that participants in the stop-group would consume less food overall than participants in the double-response group, but that this effect would be stronger for the food associated with stopping (Houben, 2011). Finally, due to the above concerns that double-response training might increase food consumption, we added a second control group, who was instructed to ‘ignore’ the signals and simply perform standard go responses throughout. We predicted that this ‘ignore’ group would show levels of food consumption intermediate between the stop and double-response groups, but that stop-training would still cause a significant reduction in intake relative to this ignore group.

### Methods and materials

#### Participants

One hundred and seventy participants (124 women) were recruited from the student and staff population at Cardiff University, using online advertising and the Psychology department's online experimental management system. Participants were aged 18–49 years and were semi-randomly assigned to groups keeping age, gender, and time seen (between 1 and 6 pm) balanced between groups.

#### Apparatus and task

Details of the apparatus are identical to those reported above for Experiment 1. The SST was the same, except for the following modifications: This version consisted of 8 blocks of 64 trials and a stop/double signal (bold frame) was presented on 25% of trials in each block (128/512 trials overall).

The signal was the same rectangular bold frame as in Experiment 1. Participants in the stop group were instructed to withhold their response, whilst participants in the double-response group were instructed to make an additional response (press the spacebar) on signal trials. Participants in the ‘ignore’ control group were simply instructed to respond to left/right location throughout and to ignore the bold frame.

Pictures from the following categories were presented in a random order: Crisps (8 exemplars); chocolate (8 exemplars); pasta (8 exemplars); pancakes (8 exemplars); non-food items (household/garden objects and clothes e.g. clock, rake, gloves; 32 exemplars). Each exemplar (64 in total) was presented 8 times (once per block). Participants received a 30 second break after every two blocks. A stop/double signal was presented on 25% of trials; however, signals were associated with the different categories of pictures as follows (see Fig. 2): Nearly half of signals (56/128; 43.75%) occurred during the presentation of specific food pictures (crisps or chocolates, counterbalanced across participants), with a minority of signals (8/128; 6.25%) occurring during the “other” food (crisp or chocolate). Therefore, one of the subsequently-eaten foods was nearly always (87.5%) associated with signals (the ‘signal food’), whilst the other food was rarely (12.5%) associated with signals (the ‘no-signal go food’). The remaining signals (64/128; 50%) occurred during the presentation of other food pictures (pasta and pancakes), which were associated with signals 50% of the time. Overall, food pictures (as a category) were associated with signals on 50% of trials. The non-food images were never associated with signals and were always standard go trials. This weighting of signals to stimulus categories was designed to encourage associative learning of food, and in particular a specific food, with response-inhibition, whilst maintaining task difficulty and attention. The stop/double signal delay (SSD) was initially set at 250 ms and continuously adjusted according to a ‘simulated’ tracking procedure as described above for Experiment 1.

#### Procedure

The procedure was identical to that described above, except for the inclusion of a taste test and provision of two foods instead of one. As before, participants were instructed to refrain from eating for three hours before the start of the study to ensure that they all arrived with “similar levels of glucose deprivation”. Upon arrival, participants gave written informed consent, and completed the same two state measures of hunger (visual analogue scale) and mood state (PANAS) as before. Participants then performed the SST, which lasted approximately 20 minutes. After task completion, participants were asked to complete a taste test (based on Houben, 2011) and the same filler questionnaires as in Experiment 1. The bogus taste test presented participants with two bowls of food – crisps (100 g; Tesco Ready Salted, 5.45 kcal/g) and chocolate buttons (210 g; Tesco Milk Chocolate Buttons, 5.4 kcal/g); these quantities were selected because they appeared as similar portions when presented in two medium-sized bowls. In addition, no participants had eaten more than 90 g of crisps in Experiment 1, so we offered 100 g. For the taste test, participants were asked to complete a 2 page questionnaire (based on Houben, 2011) with open-ended questions asking how they would describe various different aspects of the two foods (such as sweetness, saltiness and taste), along with Likert scales measuring palatability of the two foods (on a scale of 1–10) and usual frequency of consumption. Participants were instructed to “taste as much of the products as you want, as we will throw out the food that is left over at the end of this session.” They were provided with the two bowls of snack foods, a small (8 cm) glass of water and the pack of taste test and personality questionnaires. They were informed that they would be left alone for 20 minutes to taste the products and complete the questionnaires, but that extra time could be provided to complete the questionnaires at the end.

After 20 minutes any completed questionnaires were collected, participants were given the remaining eating-related questionnaires to complete as before (DEBQ and FCT), and the foods were removed and weighed in another room. The grams of crisps and chocolate consumed were converted to a common scale of kcal and analysed separately for signal and non-signal foods. At the end of the experiment all remaining questionnaires were collected and participants were asked the same series of funnelled debrief questions as in Experiment 1. Participants' height and weight was measured in order to calculate Body Mass Index (kg/m^2^). They were then debriefed and paid £6.

### Statistical analyses

Prior to analysis, data from outliers, those with missing data and participants with relevant previous experience were removed as for Experiment 1. These exclusions (n = 34; see Supplementary Table S4) resulted in a final sample of 136 in Experiment 2 (n = 44 in the inhibition condition, n = 46 in the double response condition and n = 46 in the ignore control condition). These final sample sizes resulted in 80% power to detect between-subjects pairwise differences of d ≥ 0.59, and within-subjects differences of dz ≥ 0.43.

Consumption of signal and non-signal foods (kcal) was compared using a mixed within- and between-groups ANOVA. Given uncertainty in expected effect sizes, we adopted an interim analysis approach in which a flexible stopping rule was applied with appropriate Type I error (α) correction (Strube, 2006).^4^ Thereafter, all significance tests in Experiment 2 were evaluated at the appropriate corrected α level (.0362).

A moderated regression analysis examined relationships between training condition (coded as two dummy variables), dietary restraint (DEBQ) and calorie intake. Training condition (stop or other) was the focal predictor variable, dietary restraint was the moderator variable, and calorie intake from signal foods was the dependent variable. The effect of control training (double vs. ‘ignore’) was entered into the regression model as an additional (dummy-coded) predictor variable.

### Results

The groups were well matched for age (*M* = 24.12, *SD* = 6.3), sex (73.5% female), BMI (*M* = 23.5, *SD* = 4.15; range 17.3–44.3), dietary restraint (*M* = 2.79, *SD* = 0.95), trait food craving (*M* = 62.98, *SD* = 18.96), hours since last food consumption (*M* = 5.33, *SD* = 3.9) and state measures (hunger and mood) (all p's > .4, except for negative mood, p = .09). Full descriptive information and significance tests between groups are provided in Supplementary Table S2.

Performance on standard go trials was high in all groups but was higher in the ignore relative to stop-group (Supplementary Table S2). Mean RT on correct go trials was faster in the double-response (*M* = 445.23, *SD* = 79.1) and ignore- (*M* = 406.13, *SD* = 68.68) relative to the stop-group (*M* = 604.9, *SD* = 142.94), again consistent with slowing in the stop context. Accuracy on signal trials was highest in the ignore group followed by the double-response group and the stop-group (Supplementary Table S2), again suggesting that it was easier to execute a standard or additional response than to stop an ongoing response.

**Fig. 4:**
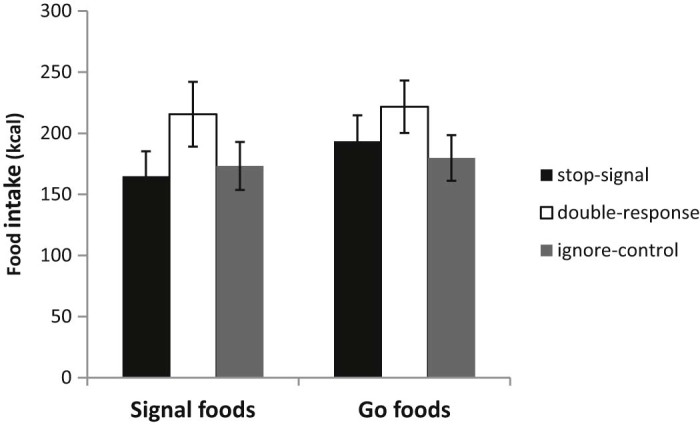
Consumption of foods associated with either a stop-/double-response (signal foods) or a standard go response (non-signal go foods) in individuals performing a food-related stop, double-response or ‘ignore’ control training task. Graphs display group mean intake of foods ± 1 standard error.

The mean calorie intake of signal and non-signal foods in each training group is shown in Fig. 4. The double-response group showed the highest calorie intake of both foods but there were no reliable overall differences between groups (F(2,133) = 1.64, p = 0.2, η^2^p = 0.024). There was no significant within-subjects effect of food type (signal or non-signal) (F(1,133) = 1.21, p = 0.27, η^2^p = 0.009) and no significant interaction between food type and group (F(2, 133) = 0.348, p = 0.71, η^2^p = 0.005).^5^ Even though the main effect of training was not significant, we contrasted the stop- and double-response group directly to allow a comparison with Experiment 1. There was no significant difference in consumption of either the signal food (t(88) = 1.51, p = .13, Cohen's d = 0.32) or the non-signal food (t(88) = 0.94, p = .35, Cohen's d = 0.2), indicating a failure to replicate the main effect of training in Experiment 1.

**Fig. 5:**
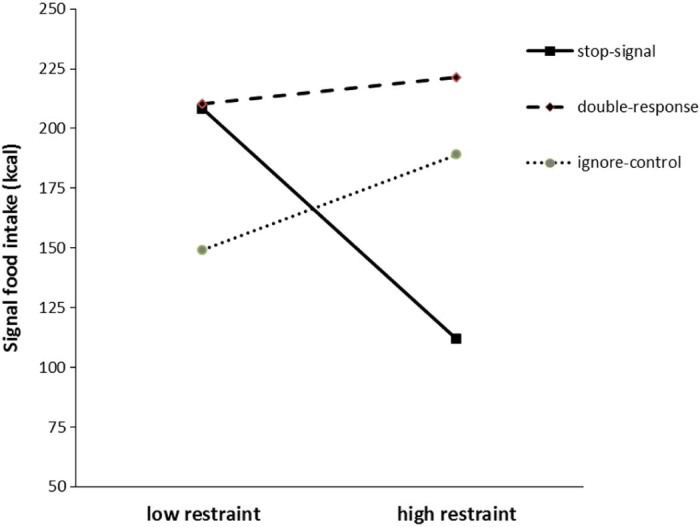
The interaction between dietary restraint and inhibition training on food intake. The plot shows the estimated mean consumption (in kcal) of the signal-associated food in each group as a function of dietary restraint (estimated at 1 SD below or above the sample mean).

A moderated regression analysis indicated that dietary restraint interacted with the effects of training on signal food consumption (t = −2.09, p = .0386; Δ R^2^ = .032); this effect was marginally significant at our level corrected for peeking (p = 0.036; see footnote 5). To understand this interaction, follow-up tests examined the main effect of training (group) at low and high levels of dietary restraint (means estimated at 1 SD below and 1 SD above the sample mean restraint score; see Fig. 5). These tests indicated no effect of training condition at low levels of restraint (F(2, 129) = 1.05, p = .35, η^2^p = 0.016) but trends at high levels of restraint (F(2, 129) = 2.85, p = .061, η^2^p = 0.042). Pairwise tests at high levels of restraint indicated less signal food consumption (*M* = −109.5 kcal, ~ 50% less) in the stop- relative to the double-response group (p = 0.02, Cohen's d = 0.5, 95% CI of the difference between stop- and double-response = −17.35 to −201.66 kcal) and a non-significant reduction (*M* = −77.1 kcal) for stop relative to the ignore-control group (p = 0.098, Cohen's d = 0.35, 95% CI of the difference between stop- and ignore-control = 14.4 to −168.65 kcal). There was no significant difference in signal food intake between ignore-control and double-response groups at high levels of restraint (p = 0.46, *M* = −32.39 kcal, Cohen's d = 0.16, 95% CI of the difference between ignore- and double-response = 53.52 to −118.29 kcal) (see Fig. 5).

There were no differences between groups in the palatability ratings (out of 10) given during the taste test to either signal (*M* = 7.18, *SD* = 2.18) or non-signal foods (*M* = 7.2, *SD* = 2.05; Supplementary Table S2). Furthermore, dietary restraint did not interact with the effects of training on palatability ratings for signal foods (t = −0.33, p = .74; Δ R^2^ < .001), suggesting that palatability ratings did not play a role in training effects on food intake.

We examined whether inhibition accuracy to signal food in the stop-group improved over time and whether this was associated with food consumption in the taste test. There was an increase in the proportion of successful food-stop trials from early (first two blocks; *M* = 0.59, *SD* = 0.244) to late (last two) blocks (*M* = 0.7, *SD* = 0.285; t(43) = 2.95 p = .005), consistent with learning the food-stop associations. However, there was no significant correlation between consumption of the signal food in the taste test and either overall signal food-stop accuracy (r(44) = −.08, p = .61) or the improvement in signal food-stop accuracy from early to late blocks (r(44) = .12, p = .44).

### Awareness of stimulus-specificity of the training task

When debriefed, the majority of participants (74%) reported noticing that the signals were associated with pictures of food, and this was higher in the stop and double-response groups than in the ignore group (Supplementary Table S2). Fewer participants (16%) reported an association between signals and images of their specific ‘signal’ food (crisps or chocolate), and this proportion was similar across the three groups (χ^2^ (2, 136) = .51, p = .77).

As in Experiment 1, no participants guessed that the aim of the study was to examine the effect of stop-training on reducing subsequent food consumption. Instead, the majority of participants (60%) thought that the food images in the task made them feel hungrier/eat more, whilst 40% did not think that the task influenced how much they snacked afterwards, and this was similar across groups (Supplementary Table S2).

### Discussion

In contrast to our first experiment, this second experiment showed no reliable main effects of training on food intake. However, the present study found a marginally significant (at corrected levels) moderation of training effects by dietary restraint. Pairwise tests indicated that individuals high in dietary restraint showed significant effects of stop- relative to double-response training in reducing intake of signal foods, consistent with the two previous reports showing a similar interaction between stimulus-specific training effects and restraint (Houben & Jansen, 2011; Veling et al., 2011).

The addition of a second ‘ignore’ control condition in this experiment failed to conclusively establish whether food-associated stop training was effective in reducing intake, or whether the double-response control condition was increasing it. Overall there were no significant differences in food consumption between any of the groups, and the reduction in restrained eaters undergoing stop- relative to ignore-training was not reliable. This finding is inconsistent with our hypothesis that the stop-group would consume less signal food than both ignore and double-response groups. It is possible that exposure to pictures of tasty, high calorie foods (which were associated with standard ‘go’ responses on 50% of ‘food’ trials in all three groups) made all participants in this experiment more disinhibited towards food than in Experiment 1, where foods were only associated with ‘go’ responses on 12.5% of trials. This greater inconsistency may have counteracted and diminished the main effect of stop- training in Experiment 2, making this effect weaker than that observed in Experiment 1 (Cohen's d for stop- vs. double-response training was 0.32 in Experiment 2 vs. 0.56 in Experiment 1). In Experiment 3 we therefore examined whether stop- vs. double-response training to non-food pictures resulted in less food consumption, which would support ‘inhibitory spillover’ effects between domains (Berkman et al., 2009; Tuk et al., 2011; Verbruggen et al., 2012), and remove any unwanted food cue exposure effects.

## Experiment 3 – effects of stop- and double-response training to non-food stimuli on consumption of two foods

The third experiment was a partial repeat of Experiment 2 (double-response and stop groups) but used only non-food pictures throughout. If general inhibition training reduces snack food intake, this could have greater therapeutic potential as it avoids exposing individuals to images of tempting foods. This experiment consisted of three experimental groups; one group received general stop-training (with no association between signals and a specific category of images), one group received stimulus-specific stop-training (signals associated with one category of non-food images, to match the stimulus-specific associations in Experiment 2) and the third group received stimulus-specific double-response training. In the general stop-training condition, stop-signal delay was dynamically adjusted and the stimulus-stop mapping was inconsistent; it is generally assumed that this version of the task involves top-down response inhibition. However, when the stimulus-stop mappings are consistent (as in Experiment 2), response inhibition may become automatised (Verbruggen, Best, Bowditch, Stevens, & McLaren, 2014; Verbruggen & Logan, 2008a). Therefore, in Experiment 3 we also included a stimulus-specific stop-training condition with neutral stimuli that were consistently associated with stopping to allow a more direct comparison between Experiments 2 and 3. After training, participants completed the same taste test as in Experiment 2. Following previous research showing significant increases in food intake in individuals given general ‘disinhibition’ training (Guerrieri et al., 2009, 2012), we predicted increased overall food intake in participants in the double-response group relative to the two stop groups.

### Methods and materials

#### Participants

One hundred and seventy participants (128 women) were recruited from the student and staff population at Cardiff University, using online advertising and the Psychology department's online experimental management system. Participants were aged 18–50 years and were semi-randomly assigned to groups keeping age, gender, and time seen (between 1 and 6 pm) balanced between groups.

#### Apparatus and task

Details of the apparatus are identical to those reported above for Experiments 1 and 2. The SST was the same as in Experiment 2, except that this version did not include any food pictures. Instead pictures were of a variety of non-food items, including the same 32 non-food pictures as in Experiment 2, along with 32 new non-food images. These additional images belonged to categories in the same way as the food images in Experiment 2, so that they could precisely replace the food images. These categories were: stationery (8 exemplars), pens (8 exemplars), electrical goods (8 exemplars) and wooden furniture (8 exemplars). As in Experiment 2, there were 8 blocks of 64 trials and a stop/double signal (bold frame) was presented on 25% of trials (128/512 trials overall).

In the general-stop group the SST task contained no association between any pictures and stop signals. In the stimulus-specific stop- and double-response groups, one category of pictures (stationary or pens, counterbalanced) was nearly always (87.5% of trials) associated with a signal. The other category (stationary or pens) was associated with a signal 12.5% of the time. The remaining signals (50%) occurred during the presentation of the other new non-food pictures (electrical goods and wooden furniture), which were therefore associated with signal trials 50% of the time. This reproduced the association between signals and the different categories of food images in Experiment 2 (see Fig. 2). As in Experiment 2, the remaining 32 non-food images were never associated with signals and were always standard go trials. This weighting of signals to stimulus categories was designed to encourage associative learning (between particular non-food items and response-inhibition), whilst maintaining the same levels of task difficulty and attention as in Experiment 2.

In the stimulus-specific training conditions, the stop/double signal delay (SSD) was initially set at 250 ms and continuously adjusted according to a ‘simulated’ tracking procedure as described above for Experiment 1. In the general stop-training condition, we used a standard tracking procedure: SSD decreased by 25 ms when a subject responded on a stop-signal trial, but increased by 25 ms when they successfully stopped (Logan, Schachar, & Tannock, 1997; Verbruggen & Logan, 2009b).

#### Procedure

The procedure was identical to that described above for Experiment 2. The only modifications were the images presented during the SST and the fact that in Experiment 3, 48 participants completed the study in exchange for course credit and 122 in exchange for the same remuneration as in Experiments 1 and 2 (£6).

### Statistical analyses

Prior to analysis, data from outliers, those with missing data, and participants with relevant previous experience were removed as for Experiments 1 and 2. These exclusions (n = 24, see Supplementary Table S4 for details) resulted in a final sample of 146 in Experiment 3 (n = 51 in the double-response condition, n = 47 in the stimulus-specific stop condition, and = 48 in the general-stop condition). These final sample sizes resulted in 80% power to detect between-subjects pairwise differences of d ≥ 0.58.

Consumption of chocolate and crisps (total kcal; there were no ‘signal’ and ‘no signal’ foods in this experiment) was compared using a between-groups ANOVA, with α = .05. A moderated regression was conducted to examine relationships between training condition (coded as one dummy variable), dietary restraint (DEBQ) and total calorie intake. Training condition (stop or other) was the focal predictor variable, dietary restraint was the moderator variable, and total calorie intake was the dependent variable. For the purpose of this analysis, therefore, both inhibition groups (stimulus-specific and general) were treated as one stop-training group.

### Results

The groups were well matched for age (*M* = 23.5, *SD* = 6.1), sex (76% female), BMI (*M* = 22.94, *SD* = 4.02, range 16.73–40.57), dietary restraint (*M* = 2.66, *SD* = 1.0), trait food craving (*M* = 65.62, *SD* = 19.93), hours since last food consumption (*M* = 5.38, *SD* = 4.03) and state measures (hunger and mood) (all p's > .28). Full descriptive information and significance tests between conditions are provided in Supplementary Table S3.

Performance on standard go trials was higher in the double- relative to stop-groups (Supplementary Table S3; post-hoc tests show both p < .001). Mean RT on correct go trials was faster in the double (*M* = 426.55, *SD* = 71.6) relative to both stop groups (stimulus-specific stop *M* = 794.08, *SD* = 175.95; general stop *M* = 755.42, *SD* = 162.74; both p < 0.001), consistent with increased cautiousness in the stop context. Accuracy on signal trials was highest in the double group, followed by the stimulus-specific stop and then the general-stop group (Supplementary Table S3; all pairwise tests p < .001), again suggesting that it was easier to execute an additional response than to stop an ongoing response.

The total consumption of crisps and chocolate was similar in the three training groups: General stop (*M* = 420.47, *SD *=* *236.53), stimulus-specific stop (M = 412.09 ± 314.04) and stimulus-specific double-response (*M* = 414.02, *SD *=* *246.22); there was no effect of group (F(2, 145) = .013, p = 0.987, η^2^p < 0.001). The mean pairwise differences in total intake (and 95% CI for the difference) were; between stimulus-specific and general stop groups *M* = −8.4 kcal (99.94 to −116.7 kcal), between stimulus-specific stop and double-response, *M* = −1.93 kcal (104.8 to −108.66 kcal), and between general-stop and double-response, *M* = 6.45 kcal (112.6 to −99.7 kcal). The moderated regression analysis showed no interaction between training condition and restraint on total intake (t = 1.08, p = .28; Δ R^2^ = .0081).^6^ Therefore in this experiment, there was no significant main effect of training condition on calorie intake and no significant moderation of the training effect by individual differences in dietary restraint. It is worth noting that mean total intake (kcal) in this Experiment 3 (*M* = 415.52, *SD* = 265.21) was similar to the total intake of the same two (signal + non-signal) foods in Experiment 2 (which varied from *M* = 358.24, *SD* = 237.69 in the stop group to *M* = 424.77, *SD* = 255.98 in the double-response group).

There were no significant differences between groups in the palatability ratings (out of 10) given during the taste test to either crisps (*M* = 6.84, *SD* = 2.09) or chocolate (*M* = 6.99, *SD* = 2.32; Supplementary Table S2).

### Awareness of stimulus-specificity of the training task

When debriefed, only a small minority of participants (8.4%) reported noticing that signals were associated with specific pictures/categories of non-food objects, and this was similar in all three groups (Supplementary Table S3). No participants guessed that the aim of the study was to examine the effect of stop-training on reducing subsequent food consumption and in contrast to Experiments 1 and 2, the majority of participants in Experiment 3 (85%) did not think that the task made them feel hungrier/eat more (as all images were of non-foods), whilst 15% did think that the task may have made them eat more (due to boredom/fatigue). This distribution of responses was similar across groups (Supplementary Table S3).

### Discussion

The lack of a difference in food intake between stop- and double-response groups in this experiment suggests two related findings: Double-response training to non-food pictures need not prime general disinhibition, and general (non-food) inhibition training need not reduce food intake. The lack of increased food intake following double-response training contrasts with previous reports of increased food intake following similar behavioural impulsivity training (Guerrieri et al., 2009, 2012). On the other hand, the lack of a general inhibition-training effect on consumption is consistent with previous reports (Guerrieri et al., 2012; Jones & Field, 2013) and supports the idea that such training needs to be stimulus-specific, perhaps involving stimulus devaluation (Houben et al., 2012a; Veling et al., 2013a) rather than a general strengthening of inhibitory control. These findings, therefore, suggest that future development of response-inhibition training for reducing overeating may benefit by focusing on food stimulus-specific training. Similarly, the lack of a general disinhibition effect in the double-response group suggests that any increased food intake in the double-response groups in Experiments 1 and 2 may have resulted from stimulus-specific food-approach training, similar to that reported by Schonberg et al. (2014).

Finally, there was no moderation of training effects by dietary restraint in this experiment, consistent with a previous study using a similar behavioural induction of general impulsivity vs. inhibition (Guerrieri et al., 2009). Instead, current dieting, rather than restraint, may moderate (reduce) the effects of general behavioural impulsivity training on food intake (Guerrieri et al., 2009).

## General discussion

The findings from our first experiment demonstrated that an unselected sample of individuals ate less following food-associated stop- vs. double- response training. Our second experiment suggested that more stimulus-specific food stop- vs. double-response training effects were only significant in restrained eaters. In the third experiment we removed exposure to pictures of food in the task, which we thought may be counteracting the inhibition-training effect. However, there was no effect of non-food stop- vs. double-response training, suggesting that food stimulus-specific training is required for effects on intake to be observed.

### Stimulus-specificity of training effects

The aim of this research is to develop an intervention to help people control their eating behaviour. Our findings and others' suggest that such an intervention should involve food stimuli. Furthermore, the strongest training effects may result from more consistent associations between ‘food’ as a category and inhibition signals. Whilst the comparison of training effects across studies and research groups is complicated by the different tasks (go/no-go vs. stop-signal), food intake measures and samples used (e.g. our samples were more heterogeneous and may have had more varied food preferences than in previous studies), we attempt to draw some conclusions. As discussed above, our first experiment contained more consistent associations between all food pictures and stopping (87.5%) than our second experiment (50% overall 'food'-stop associations), which may have contributed to the stronger main effects of training observed in Experiment 1. Some previous studies have used consistent (100%) associations between ‘food’ and inhibition (Van Koningsbruggen et al., 2014; Veling et al., 2011) and have demonstrated main effects of food no-go training (albeit on a different measure, portion size; Van Koningsbruggen et al., 2014) as we did in Experiment 1, whilst others have used consistent (100%) associations for specific foods but lower overall associations (50–66%) between ‘food’ and inhibition, and have only observed effects in impulsive or restrained individuals (Houben, 2011; Houben & Jansen, 2011), as we did in Experiment 2. Taken together with our results, these findings suggest that the consistency of associations between ‘food’ and inhibition, as well as between specific foods and inhibition may influence whether main effects, or moderated effects, of training are observed.

The complexity and difficulty of food response-inhibition tasks varies considerably between studies. Some have employed very brief (72 trials), simple and easy go/no-go tasks (Veling et al., 2011) whilst others, like our Experiment 2, have employed much longer (512 trials) and more demanding tasks. The number of different food and filler pictures presented has varied from 8 (Veling et al., 2011) to 64 (our Experiment 2), the total number of ‘food’-inhibition trials has varied from 12 (Veling et al., 2011) to 160 (Houben & Jansen, 2011), and the number of times each signal food picture (exemplar) was associated with an inhibition-signal has varied from 4 (Veling et al., 2011), to 7–9 (our experiments), to 12 (Houben, 2011), and 20 (Houben & Jansen, 2011). These considerable differences in training tasks have resulted in fairly similar results across studies, consistent with evidence that systematically increasing the number of food-no-go pairings from 4 to 12 to 24 does not modify training effects (on food evaluation; Veling et al., 2013a). It is possible that brief tasks involving very few food-signal pairings recruit different mechanisms (e.g. stimulus devaluation; Veling et al., 2013a) compared to longer tasks that include more food-signal pairings (which may also recruit automatic inhibition; Verbruggen & Logan, 2008a). The longevity of training effects may also depend on the amount of training. Future research is required to systematically examine this idea in order to determine optimal training task parameters for use in applied settings.

In terms of participants' self-reported awareness of the stimulus-specificity of training, the majority of participants in the stop- and double-response groups in Experiments 1 and 2 noticed the ‘food’-signal associations. Whilst it is unclear how much can be inferred from debrief interviews conducted at the end of experiments (Newell & Shanks, 2014), it is interesting that reported awareness was not lower in Experiment 2 relative to 1 despite the greater overall inconsistency in ‘food’-signal associations. In contrast, very few participants reported the signal associations with non-food pictures in Experiment 3. It may have been harder for participants to discriminate between distinct categories of non-food items in Experiment 3 than to discriminate between food and non-food categories in Experiments 1 and 2. The increased attentional and motivational salience of food pictures (Hardman, Rogers, Etchells, Houstoun, & Munafò, 2013) could also have enhanced learning and awareness of the associations between signals and food pictures in Experiments 1 and 2.

### Control conditions

In nearly all previous lab studies reporting effects of food or alcohol response-inhibition training on consumption, the effects have been compared to control conditions requiring either go or inconsistent go/stop responses to food or alcohol pictures (Bowley et al., 2013; Houben, 2011; Houben et al., 2012a; Houben & Jansen, 2011; Houben et al., 2011; Veling et al., 2011). This was also the case in the present study. The concern is that these control conditions, by exposing participants to tempting cues and requiring them to make a response at least half of the time, could be encouraging participants to ‘approach’ the foods and consume more (Bowley et al., 2013; Schonberg et al., 2014), making the true effects of inhibition training hard to quantify. Our findings in Experiment 2, showing significant differences in intake between restrained eaters in the stop- vs. double-response but not vs. the ignore-control group suggest this is a valid concern. Moreover, one cannot assume that control conditions that employ inconsistent pairing of food stimuli with go and stop responses (Houben, 2011; Houben & Jansen, 2011) constitute a neutral baseline because evidence suggests that inconsistent reinforcement can increase attention to, and motivational salience of, conditioned stimuli (Anselme, Robinson, & Berridge, 2013; Pearce & Hall, 1980).

One solution to this potential confound is to compare food response-inhibition training effects to an additional neutral (non-food) inhibition condition, and there are now two studies indicating inhibition-training effects for alcohol and food relative to such a conservative control group (Jones & Field, 2013; Veling, Van Koningsbruggen, Aarts, & Stroebe, 2014). Another solution is to conduct within-subjects repeated-measures studies, with intake or other critical dependent variables (such as body weight) monitored at baseline and after inhibition training, to measure changes from the participants' own baseline and aid interpretation of training effects (Houben et al., 2012a; Houben & Jansen, 2011; Houben et al., 2011; Lawrence et al., 2014; Veling et al., 2014). Obviously a control group (ideally involving no cue exposure) is still required to control for any changes over time. Such mixed, longitudinal study designs will help to conclusively establish genuine effects of food and alcohol response-inhibition training on behaviour.

### Individual differences

Our second experiment indicated significantly less signal food intake following stop vs. double-response training in restrained eaters (Houben & Jansen, 2011; Veling et al., 2011). There was no interaction between restraint and training effects in Experiments 1 and 3 despite similar levels of restraint in all three experiments (Supplementary Tables S1–S3). These different effects of dietary restraint may have resulted from methodological variations between experiments.

First, the interaction with dietary restraint on signal food intake in Experiment 2 but not 3 suggests that restraint only affects food stimulus-specific response-inhibition training (Houben, 2011; Veling et al., 2011). This is consistent with suggestions that stop-training is only effective when strong impulses or approach tendencies are evoked in the first place (Houben, 2011; Veling et al., 2011), such as when palatable food cues are presented to restrained eaters (e.g. Fedoroff et al., 1997; Veenstra & de Jong, 2010).

Second, the interaction between training effects and dietary restraint on food intake in Experiment 2 but not 1 could be linked to the increased number of foods offered and the addition of the taste test in Experiment 2. These factors increased the amount and variance of overall food intake from *M* = 149.6, *SD* = 110.2 kcal in Experiment 1 to *M* = 379, *SD* = 239.35 kcal in Experiment 2, consistent with the known effects of food variety and taste-test priming manipulations in promoting consumption (Cornell, Rodin, & Weingarten, 1989; Rolls et al., 1981). The taste test may also have enhanced the effect of individual differences in dietary restraint on intake by forcing restrained eaters to ‘break their diet’ and consume high-calorie foods (‘counter-regulation’, Herman & Mack, 1975), particularly following double-response (disinhibition) training. In other words, the taste test and double-response training may have had additive effects on increasing disinhibition towards food, particularly in restrained eaters. In sum, the increased amount and variance of food intake in Experiment 2 may have made it harder to detect a main effect of stop- vs. double-response training but easier to detect the moderating influence of dietary restraint. Whilst the restraint scale used here (DEBQ; Van Strien et al., 1986) differs from that used previously (Restraint Scale; Herman & Polivy, 1980), these findings further suggest that food response-inhibition training may be particularly effective in individuals who try (but may struggle) to restrict their food intake. However, if the moderating effects of restraint in this and previous laboratory studies are partly driven by experimental manipulations (disinhibition in the control groups), one should be wary of suggesting that only restrained eaters would benefit from food response inhibition training in the real world. In Experiment 1, where less disinhibition was primed (i.e. there was no taste test), main effects of training were observed so it would be worth further investigating training effects in all individuals whilst continuing to consider individual differences in restraint. Future studies should also consider individual differences in other potential moderators of training effects that are associated with ‘impulsive’ eating, such as BMI (Veling et al., 2014), snacking habits (Veling et al., 2013b) and past success at weight control (Houben et al., 2012b).

### Limitations

Across all studies, task performance data showed that participants in the double-response and ignore-control groups were faster and more accurate than those in the stop-groups indicating that it was easier to execute an additional response than to stop an ongoing response. The results of Experiment 3 also showed that it was easier to stop a response if the signals had been consistently associated with a stimulus category than it was to stop a response to random stimuli, which further confirms that participants had learned the stimulus-stop associations (Verbruggen & Logan, 2008a). Task difficulty was therefore unequal between the training conditions, increasing in mental effort from the ignore- to double-response to stop tasks. The increased mental effort (and potential ego-depletion) in the stop-groups may have promoted subsequent food intake (Boon, Stroebe, Schut, & Ijntema, 2002; Gailliot & Baumeister, 2007), potentially counteracting the effects of stop-training in our experiments. Several previous response-inhibition training studies have used the Go/No-go task rather than stop-signal task (Houben & Jansen, 2011; Houben et al., 2011; Veling et al., 2011), which is believed to recruit mechanisms of action restraint rather than action cancellation (Schachar et al., 2007). The Go/No Go task is simpler and easier to execute than the stop-signal task and may be more appropriate for use in studies of response-inhibition training for several reasons.

First, the relative ease of food no-go tasks reduces confounds associated with task difficulty such as the ego-depletion effects discussed above. Second, the higher level of inhibition accuracy on food no-go (relative to stop-signal) trials may facilitate the learning of associations between foods and response inhibition: It is possible to achieve 100% successful stopping to foods that are paired 100% of the time with no-go signals (and no-go responses) in Go/No Go tasks, which should increase associative learning relative to the partial reinforcement (catch or unsuccessful stop trials) in stop-signal tasks (Le Pelley & McLaren, 2003; see also Verbruggen & Logan, 2008a, for a brief discussion of possible effects of the outcome of the stop process). These factors, which promote stimulus-specific associative learning of response inhibition, may be important for the development of the two main mechanisms proposed to underlie training effects; automatic inhibition (Verbruggen & Logan, 2008a; Verbruggen et al., 2014, Veling et al., 2011; cf Houben et al., 2012a) and stimulus devaluation (Houben et al., 2012b; Veling et al., 2013a; cf Bowley et al., 2013). On the other hand, if strengthening controlled (rather than automatic) inhibition is important for training effects then more challenging stop-signal tasks that require action cancellation may be more effective. The present studies were not designed to investigate the mechanisms underlying training effects and our findings do not therefore directly support any of the above mechanisms. However, our results do indicate that a strengthening of inhibitory control is unlikely to explain training effects on reducing food intake; there were no effects of general stop-training on intake (Experiment 3) and, whilst participants in the food-stop groups did learn to inhibit to food over training (Experiments 1 and 2), neither the mean food-stop-accuracy or the improvement in stopping to food (learning) was correlated with intake. Ultimately, whether food-associated Go/No Go or stop-signal training tasks are more effective in reducing calorie intake is an empirical question: We have recently examined this in a separate study, which suggested that Go/No Go training is more effective (Adams et al., in preparation).

The methodological differences between the current experiments prevented us from comparing their results statistically. We have compared them in descriptive terms but did not compare them directly due to differences in the number of foods provided and whether a taste test was included (Experiment 1 vs. Experiments 2 and 3), and whether one food was strongly associated with signals (Experiment 2 vs. Experiment 3).

Finally, whilst the majority of participants reported noticing the signal-food associations in Experiments 1 and 2, no participants guessed the true aim of the experiments so we do not believe our results can be explained in terms of demand characteristics. In fact, about half of the participants in Experiments 1 and 2 (including in the food stop-groups) thought the task *increased* their hunger and motivation to eat, whilst the other half thought it had no effect, which argues against demand characteristics explaining the stop-training effects.

In conclusion, the present findings improve our understanding of response-inhibition training for overeating. They suggest that food stimulus-specific learning is important for training effects on food intake, they highlight the potential confounds of food ‘go’ or ‘approach’ control training conditions, and reinforce existing findings that such training may not be effective for everyone – individual differences in dietary restraint may be relevant. By taking these factors into account we can further develop and test interventions to help people regain control over their eating behaviour.

## Footnotes

1 A subset of 10 participants (5 in each group) completed 10 blocks of 36 trials. These participants saw 12 fewer exemplars (the same 2 crisp pictures, 2 other food pictures and 8 non-food pictures were missing from their task). Data suggest that varying the number of food-stop pairings (from 4 to 24) does not alter training effects (Veling et al., 2013a) so data from these participants were included. However, the results with these 10 participants excluded are also reported below.

2 Results are also presented with the outliers for intake included in the analysis – see footnotes in Results.

3 Analyses were repeated excluding the 10 participants who performed a shorter variant of the training tasks. There were similar reductions in intake in the stop-group relative to the double-response group (mean difference: −63.64 kcal, 95% confidence interval (CI) = 0.99 to −128.28 kcal; F(1, 42) = 3.95, p = 0.053, η^2^p = 0.086, Cohen's d = 0.59). Dietary restraint did not interact significantly with the effect of training in the moderated regression analysis in this subset of participants (t = −.24, p = .81; Δ R^2^ = .0013).

4 Initially, data collection continued in Experiment 2 until the samples were approximately equivalent to Experiment 1 (~27 participants per group). Analysis of variance revealed no significant group differences, therefore we added an additional ~18 participants to each group to increase power. To accommodate the elevation of α due to this flexible stopping rule, a correction for ‘peeking’ was applied based on the method of Strube (2006). In brief, this approach simulates the consequences of repeatedly testing the null hypothesis before a final sample is obtained. Based on an initial sample size of (on average) 27 participants per group and a final sample size of (on average) 45 participants per group, a single peeking correction was applied with an increment of 18 participants and 10,000 iterations for simulating the true α. This revealed an estimated α of .069 (95% CI = .064–.073); we therefore applied a proportional correction of α in which α_corrected_ = α_original_ × (α_original_ ÷ α_estimated_). This equation revealed a value of α_corrected_ = .0362 [.05 × (.05 ÷ .069)].

5 This analysis was repeated including the 2 participants who scored >3SD above the group mean on intake. There were similar (non-significant) effects of group (F(2,135) = 1.7, p = 0.19, η^2^p = 0.025), within-subjects effects of food type (signal or non-signal) (F(1,135) = 0.72, p = 0.4, η^2^p = 0.005) and food type by group interaction (F(2, 135) = 0.53, p = 0.59, η^2^p = 0.008). A moderated regression analysis indicated that dietary restraint interacted with the effects of training on signal food consumption (t = −2.04, p = .044; Δ R^2^ = .03).

6 The analysis was repeated including the single participant who scored >3SD above the group mean on intake. There were similar (non-significant) effects of group on total intake (F(2,146) = 0.061, p = 0.94, η^2^p = 0.001) and no interaction between dietary restraint and training effects (t = −1.62, p = 0.11; Δ R^2^ = .018).

## References

[bib0010] Anselme P., Robinson M.J., Berridge K.C. (2013). Reward uncertainty enhances incentive salience attribution as sign-tracking. Behavioural Brain Research.

[bib0015] Aron A.R. (2011). From reactive to proactive and selective control. Developing a richer model for stopping inappropriate responses. Biological Psychiatry.

[bib0020] Batterink L., Yokum S., Stice E. (2010). Body mass correlates inversely with inhibitory control in response to food among adolescent girls. An fMRI study. Neuroimage.

[bib0025] Beaver J.D., Lawrence A.D., Van Ditzhuijzen J., Davis M.H., Woods A., Calder A.J. (2006). Individual differences in reward drive predict neural responses to images of food. Journal of Neuroscience.

[bib0030] Berkman E.T., Burklund L., Lieberman M.D. (2009). Inhibitory spillover. Intentional motor inhibition produces incidental limbic inhibition via right inferior frontal cortex. Neuroimage.

[bib0035] Boon B., Stroebe W., Schut H., Ijntema R. (2002). Ironic processes in the eating behaviour of restrained eaters. British Journal of Health Psychology.

[bib0040] Bowley C., Faricy C., Hegarty B., Johnstone S.J., Smith J.L., Kelly P.J. (2013). The effects of inhibitory control training on alcohol consumption, implicit alcohol-related cognitions and brain electrical activity. International Journal of Psychophysiology.

[bib0045] Carnell S., Kim Y., Pryor K. (2012). Fat brains, greedy genes, and parent power. A biobehavioural risk model of child and adult obesity. International Review of Psychiatry.

[bib0050] Cornell C.E., Rodin J., Weingarten H. (1989). Stimulus-induced eating when satiated. Physiology & Behavior.

[bib0055] Demos K.E., Heatherton T.F., Kelley W.M. (2012). Individual differences in nucleus accumbens activity to food and sexual images predict weight gain and sexual behavior. The Journal of Neuroscience.

[bib0060] Department of Health (2012). Facts and figs on obesity. http://www.dh.gov.uk/health/2012/04/obesityfacts/.

[bib0065] Doallo S., Raymond J.E., Shapiro K.L., Kiss M., Eimer M., Nobre A.C. (2012). Response inhibition results in the emotional devaluation of faces. Neural correlates as revealed by fMRI. Social Cognitive and Affective Neuroscience.

[bib0070] Dodds C.M., Morein-Zamir S., Robbins T.W. (2011). Dissociating inhibition, attention, and response control in the frontoparietal network using functional magnetic resonance imaging. Cerebral Cortex.

[bib0075] Fedoroff I.C., Polivy J., Herman C.P. (1997). The effect of pre-exposure to food cues on eating behaviour of restrained and unrestrained eaters. Appetite.

[bib0080] Flegal K.M. (2005). Epidemiologic aspects of overweight and obesity in the United States. Physiology & Behavior.

[bib0085] Flegal K.M., Carroll M.D., Kit B.K., Ogden C.L. (2012). Prevalence of obesity and trends in the distribution of body mass index among US adults, 1999–2010. JAMA: The Journal of the American Medical Association.

[bib0090] Flint A., Raben A., Blundell J.E., Astrup A. (2000). Reproducibility, power and validity of visual analogue scales in assessment of appetite sensations in single test meal studies. International Journal of Obesity and Related Metabolic Disorders: Journal of the International Association for the Study of Obesity.

[bib0095] Gailliot M.T., Baumeister R.F. (2007). The physiology of willpower. Linking blood glucose to self-control. Personality and Social Psychology Review.

[bib0100] Grucza R.A., Krueger R.F., Racette S.B., Norberg K.E., Hipp P.R., Bierut L.J. (2010). The emerging link between alcoholism risk and obesity in the United States. Archives of General Psychiatry.

[bib0105] Guerrieri R., Nederkoorn C., Jansen A. (2012). Disinhibition is easier learned than inhibition. The effects of (dis)inhibition training on food intake. Appetite.

[bib0110] Guerrieri R., Nederkoorn C., Schrooten M., Martijn C., Jansen A. (2009). Inducing impulsivity leads high and low restrained eaters into overeating, whereas current dieters stick to their diet. Appetite.

[bib0115] Guerrieri R., Nederkoorn C., Stankiewicz K., Alberts H., Geschwind N., Martijn C. (2007). The influence of trait and induced state impulsivity on food intake in normal-weight healthy women. Appetite.

[bib0120] Hardman C.A., Rogers P.J., Etchells K.A., Houstoun K.V., Munafò M.R. (2013). The effects of food-related attentional bias training on appetite and food intake. Appetite.

[bib0125] Hayes A.F., Matthes J. (2009). Computational procedures for probing interactions in OLS and logistic regression. SPSS and SAS implementations. Behavior Research Methods.

[bib0130] Herman C.P., Mack D. (1975). Restrained and unrestrained eating. Journal of Personality.

[bib0135] Herman C.P., Polivy J.P., Stunkard A.J. (1980). Restrained eating. Obesity.

[bib0140] Hill J.O., Wyatt H.R., Reed G.W., Peters J.C. (2003). Obesity and the environment. Where do we go from here?. Science.

[bib0145] Houben K. (2011). Overcoming the urge to splurge. Influencing eating behavior by manipulating inhibitory control. Journal of Behavior Therapy and Experimental Psychiatry.

[bib0150] Houben K., Havermans R.C., Nederkoorn C., Jansen A. (2012). Beer à no-go. Learning to stop responding to alcohol cues reduces alcohol intake via reduced affective associations rather than increased response inhibition. Addiction (Abingdon, England).

[bib0155] Houben K., Jansen A. (2011). Training inhibitory control. A recipe for resisting sweet temptations. Appetite.

[bib0160] Houben K., Nederkoorn C., Jansen A. (2012). Too tempting to resist? Past success at weight control rather than dietary restraint determines exposure-induced disinhibited eating. Appetite.

[bib0165] Houben K., Nederkoorn C., Jansen A. (2014). Eating on impulse. The relation between overweight and food-specific inhibitory control. Obesity.

[bib0170] Houben K., Nederkoorn C., Wiers R.W., Jansen A. (2011). Resisting temptation. Decreasing alcohol-related affect and drinking behavior by training response inhibition. Drug and Alcohol Dependence.

[bib0175] Inzlicht M., Schmeichel B.J., Macrae C.N. (2014). Why self-control seems (but may not be) limited. Trends in Cognitive Sciences.

[bib0180] Johnson F., Pratt M., Wardle J. (2012). Dietary restraint and self-regulation in eating behavior. International Journal of Obesity.

[bib0185] Jones A., Field M. (2013). The effects of cue-specific inhibition training on alcohol consumption in heavy social drinkers. Experimental and Clinical Psychopharmacology.

[bib0190] Lawrence N.S., Hinton E.C., Parkinson J.A., Lawrence A.D. (2012). Nucleus accumbens response to food cues predicts subsequent snack consumption in women and increased body mass index in those with reduced self-control. Neuroimage.

[bib0195] Lawrence N., Verbruggen F., Morrison S., Parslow D., O'Sullivan J., Javaid M. (2014). Training response inhibition to food to reduce overeating. Journal of Psychopharmacology.

[bib0200] Le Pelley M.E., McLaren I.P.L. (2003). Learned associability and associative change in human causal learning. Quarterly Journal of Experimental Psychology.

[bib0205] Logan G.D., Dagenbach D., Carr T.H. (1994). On the ability to inhibit thought and action. A user's guide to the stop signal paradigm. Inhibitory processes in attention, memory and language.

[bib0210] Logan G.D., Schachar R.J., Tannock R. (1997). Impulsivity and inhibitory control. Psychological Science.

[bib0215] Masicampo E.J., Baumeister R.F. (2008). Toward a physiology of dual-process reasoning and judgment. Lemonade, willpower, and expensive rule-based analysis. Psychological Science.

[bib9010] MathWorks (2011). MATLAB (version R2011b).

[bib0220] McLaren I.P., Forrest C.L., McLaren R.P., Jones F.W., Aitken M.R., Mackintosh N.J. (2013). Associations and propositions. The case for a dual-process account of learning in humans. Neurobiology of Learning and Memory.

[bib0225] Murdaugh D.L., Cox J.E., Cook I.I.I.E.W., Weller R.E. (2012). fMRI reactivity to high-calorie food pictures predicts short- and long-term outcome in a weight-loss program. Neuroimage.

[bib0230] Nederkoorn C., Braet C., Van Eijs Y., Tanghe A., Jansen A. (2006). Why obese children cannot resist food. The role of impulsivity. Eating Behaviors.

[bib0235] Nederkoorn C., Coelho J.S., Guerrieri R., Houben K., Jansen A. (2012). Specificity of the failure to inhibit responses in overweight children. Appetite.

[bib0240] Nederkoorn C., Houben K., Hofmann W., Roefs A., Jansen A. (2010). Control yourself or just eat what you like? Weight gain over a year is predicted by an interactive effect of response inhibition and implicit preference for snack foods. Health Psychology.

[bib0245] Nederkoorn C., Smulders F.T., Havermans R.C., Roefs A., Jansen A. (2006). Impulsivity in obese women. Appetite.

[bib0250] Newell B.R., Shanks D.R. (2014). Unconscious influences on decision making. A critical review. Behavioral and Brain Sciences.

[bib0255] Nijs I.M., Franken I.H., Muris P. (2007). The modified Trait and State Food-Cravings Questionnaires. Development and validation of a general index of food craving. Appetite.

[bib0260] Pearce J.M., Hall G. (1980). A model for Pavlovian learning. Variations in the effectiveness of conditioned but not of unconditioned stimuli. Psychological Review.

[bib0265] Rolls B.J., Rowe E.A., Rolls E.T., Kingston B., Megson A., Gunary R. (1981). Variety in a meal enhances food intake in man. Physiology & Behavior.

[bib0270] Schachar R., Logan G.D., Robaey P., Chen S., Ickowicz A., Barr C. (2007). Restraint and cancellation. Multiple inhibition deficits in attention deficit hyperactivity disorder. Journal of Abnormal Child Psychology.

[bib0275] Schonberg T., Bakkour A., Hover A.M., Mumford J.A., Nagar L., Perez J. (2014). Changing value through cued approach. An automatic mechanism of behavior change. Nature Neuroscience.

[bib0280] Strube M.J. (2006). SNOOP. A program for demonstrating the consequences of premature and repeated null hypothesis testing. Behavior Research Methods.

[bib0285] Tuk M.A., Trampe D., Warlop L. (2011). Inhibitory spillover. Increased urination urgency facilitates impulse control in unrelated domains. Psychological Science.

[bib0290] Van Koningsbruggen G.M., Veling H., Stroebe W., Aarts H. (2014). Comparing two psychological interventions in reducing impulsive processes of eating behaviour. Effects on self-selected portion size. British Journal of Health Psychology.

[bib0295] Van Strien T., Frijters J.E.R., Bergers J.P.A., Defares P.B. (1986). The Dutch Eating Behavior Questionnaire (DEBQ) for assessment of restrained, emotional, and external eating behaviour. The International Journal of Eating Disorders.

[bib0300] Veenstra E.M., de Jong P. (2010). Restrained eaters show enhanced automatic approach tendencies towards food. Appetite.

[bib0305] Veling H., Aarts H. (2009). Putting behaviour on hold decreases reward value of need-instrumental objects outside of awareness. Journal of Experimental Social Psychology.

[bib0310] Veling H., Aarts H., Papies E.K. (2011). Using stop signals to inhibit chronic dieters’ responses toward palatable foods. Behaviour Research and Therapy.

[bib0315] Veling H., Aarts H., Stroebe W. (2013). Stop signals decrease choices for palatable foods through decreased food evaluation. Frontiers in Psychology.

[bib0320] Veling H., Aarts H., Stroebe W. (2013). Using stop signals to reduce impulsive choices for palatable unhealthy foods. British Journal of Health Psychology.

[bib0325] Veling H., Holland R.W., Van Knippenberg A. (2008). When approach motivation and behavioral inhibition collide. Behavior regulation through stimulus devaluation. Journal of Experimental Social Psychology.

[bib0330] Veling H., Van Koningsbruggen G.M., Aarts H., Stroebe W. (2014). Targeting impulsive processes of eating behavior via the internet. Effects on body weight. Appetite.

[bib0335] Verbruggen F., Adams R., Chambers C.D. (2012). Proactive motor control reduces monetary gambling. Psychological Science.

[bib0340] Verbruggen F., Aron A.R., Stevens M.A., Chambers C.D. (2010). Theta burst stimulation dissociates attention and action updating in human inferior frontal cortex. Proceedings of the National Academy of Sciences of the United States of America.

[bib0345] Verbruggen F., Best M., Bowditch W.A., Stevens T., McLaren I.P.L. (2014). The inhibitory control reflex. Neuropsychologia.

[bib0350] Verbruggen F., Logan G.D. (2008). Automatic and controlled response inhibition. Associative learning in the go/no-go and stop-signal paradigms. Journal of Experimental Psychology. General.

[bib0355] Verbruggen F., Logan G.D. (2008). Response inhibition in the stop-signal paradigm. Trends in Cognitive Sciences.

[bib0360] Verbruggen F., Logan G.D. (2009). Proactive adjustments of response strategies in the stop-signal paradigm. Journal of Experimental Psychology. Human Perception and Performance.

[bib0365] Verbruggen F., Logan G.D. (2009). Models of response inhibition in the stop-signal and stop-change paradigms. Neuroscience and Biobehavioral Reviews.

[bib0370] Watson D., Clark L.A., Tellegen A. (1988). Development and validation of brief measures of positive and negative affect. The PANAS scales. Journal of Personality and Social Psychology.

[bib0375] Yeomans M.R. (2000). Rating changes over the course of meals. What do they tell us about motivation to eat?. Neuroscience and Biobehavioral Reviews.

