# Supplementary material for: Stopping to food can reduce intake. Effects of stimulus-specificity and individual differences in dietary restraint
Source: Appetite. 2015 Feb 1;85:91–103. doi: 10.1016/j.appet.2014.11.006 (PMC4286116; doi:10.1016/j.appet.2014.11.006)
Supplement: Appendix S1 — Tables S1–S4. [file mmc1.docx]

**Supplementary Table 1.** This table provides descriptive information of participants in each training condition in Experiment 1 with between-group significance tests.

Experiment 1

Group Stop-signal Double-response *F-*tests

N = 29 N = 25 *F*(1,53)

Age 25.07 (5.9) 22.84 (3.9) *F*=2.58, *p*=.11

Sex (% females) 18 (62%) 14 (56%)  *χ^2^*=.21, *p*=.65

BMI 22.9 (4) 22.9 (3.52) *F*<0.01, *p*=.99

Restraint (DEBQ) 2.73 (.96) 2.32 (.83) *F*=2.79, *p*=.1

Hours since last food 4.27 (2.7) 5.29 (3.6) *F*=1.39, *p*=.24

Hunger 43.2 (24.4) 44.5 (19.1) *F*=0.05, *p*=.83

Fullness 17.7 (18.4) 15.3 (17) *F*=0.25, *p*=.62

Desire to eat 47.8 (28.4) 48.5 (26.1) *F*=0.01, *p*=.92

Positive mood (PANAS) 26.3 (8.43) 27.1 (6.62) *F*=0.14, *p*=.71

Negative mood (PANAS) 11.9 (2) 12.1 (2.5) *F*=0.09, *p*=.76

Food Craving Trait 58.93 (16.77) 60.24 (18.58) *F*=0.07, *p*=.79

Accuracy on go trials 97.3 (2.6) 97.7 (1.9) *F*=0.33, *p*=.57

Accuracy on signal trials 78.6 (10.4) 95.8 (3.2) *F*=63.6, *p*<.001

Reaction time on go trials 599.5 (150.9) 424.9 (74.9) *F*=27.57, *p*<.001

Awareness of signal-food 76 90 *χ^2^=1.8, p=.18*

association (% of group)

Task increased intake 31 48 *χ^2^=1.63, p=.2*

(% of group)

**Supplementary Table 2.** This table provides descriptive information of participants in each training condition in Experiment 2 with between-group significance tests.

Experiment 2

Group Stop-signal Double-response Ignore *F*-tests

N = 44 N = 46 N = 46 *F*(2,135)

Age 24.4 (6.42) 24.8 (6.75) 23.2 (5.57)^1^ *F*=0.83, *p*=.44

Sex (% females) 33 (75%) 33 (72%) 34 (74%) *χ^2^*=0.13, *p*=.94

BMI 23.95 (4.07) 23.52 (4.82)^1^ 23.12 (3.52) *F*=0.45, *p*=.64

Restraint (DEBQ) 2.69 (0.91) 2.74 (1.04) 2.93 (0.89)^1^ *F*=0.75, *p*=.47

Hours since last food 5.68 (4.28) 4.9 (3.71) 5.42 (3.7) *F*=0.46, *p*=.63

Hunger 46.7 (19.7) 43.6 (20.2) 44.8 (17.7) *F*=0.29, *p*=.75

Fullness 17 (17.7) 14.3 (15.6) 17.7 (18) *F*=0.52, *p*=.6

Desire to eat 54.5 (18.9) 51 (23.7) ^1^ 49.3 (22) *F*=0.68, *p*=.51

Positive mood (PANAS) 25.8 (4.72) 26.9 (6.54) 25.6 (5.98) *F*=0.69, *p*=.51

Negative mood (PANAS) 12.9 (3.75) 11.9 (2.06) 13.6 (4.79) *F*=2.42, *p*=.09

Food Craving Trait 60.18 (19.4) 64.35 (17.21) 64.31 (20.3)^1^ *F*=0.71, *p*=.5

Accuracy on go trials 96.4 (4.61) 97.8 (2.57) 98.6 (1.6) *F*=5.55, *p*=.005

Accuracy on signal trials 65.86 (19.92) 91.97 (9.54) 98.96 (1.07) *F*=85.31, *p*<.001

Reaction time on go trials 604.9 (142.94) 445.23 (79.1) 406.13 (68.68) *F*=48.07, *p*<.001

Palatability crisps 7.02 (1.77) 6.8 (2.15) 6.74 (1.95) *F*=0.26, *p*=.78

Palatability chocolate 7.45 (2.16) 7.33 (2.57) 7.8 (1.88) *F*=0.57, *p*=.57

Palatability signal food 7.36 (2) 7.04 (2.47) 7.15 (2.08) *F*=0.25, *p*=.78

Palatability nonsignal food 7.11 (1.97) 7.09 (2.29) 7.39 (1.89) *F*=0.31, *p*=.74

Awareness of signal-food 93 83 48 *χ^2^*=26.7, *p*<.001

association (% of group)

Task increased intake 59 50 70 *χ^2^*=3.66, *p*=.16

(% of group)

^1^Data missing from one participant in this group

**Supplementary Table 3.** This table provides descriptive information of participants in each training condition in Experiment 3 with between-group significance tests.

Experiment 3 (Non-food stimuli)

Group Stimulus-specific Stimulus-specific General

Stop-signal Double-response Stop-signal *F*-tests

N = 47 N = 51 N = 48 F(2,145)

Age 24.6 (5.88) 23.1 (6.77)^1^ 22.8 (5.51) *F*=1.27, *p*=.28

Sex (% females) 33 (70%) 39 (76%) 39 (81%) *χ^2^*=1.6, *p*=.45

BMI 22.95 (4.28) 23.45 (4.06)^1^ 22.4 (3.72) *F*=0.83, *p*=.44

Restraint (DEBQ) 2.59 (0.87) 2.56 (1.09)^1^ 2.82 (1.01) *F*=1.01, *p*=.37

Hours since last food 5.67 (4.14)^1^ 5.54 (3.73)^2^ 4.95 (4.25) *F*=0.43, *p*=.65

Hunger 40.6 (20.9) 45.9 (19.1)^1^ 47 (22.3) *F*=1.3, *p*=.28

Fullness 18.9 (19.4) 14.4 (15.3)^1^ 19.2 (16.7) *F*=1.18, *p*=.31

Desire to eat 50 (24) 53.2 (25.9)^1^ 54.4 (21.6) *F*=0.42, *p*=.66

Positive mood (PANAS) 25.7 (6.1) 24.8 (6.52) 25.1 (6.38) *F*=0.24, *p*=.79

Negative mood (PANAS) 12.96 (4.02) 12.4 (2.42) 12.92 (3.46) *F*=0.41, *p*=.66

Food Craving Trait 67.4 (19.94) 63.06 (19.27)^1^ 66.54 (20.74) *F*=0.65, *p*-.52

Accuracy on go trials 91.21 (8.59) 98.16 (1.59) 89.99 (10.54) *F*=15.8, *p*<.001

Accuracy on signal trials 64.96 (11) 93.03 (6) 57.31 (8) *F*=242.8, *p*<.001

Reaction time on go trials 794.08 (175.95) 426.55 (71.6) 755.42 (162.74) *F*=99.1, *p*<.001

Palatability crisps 6.61 (2.02)^1^ 7.08 (2.23) 6.81 (2.04) *F*=0.61, *p*=.54

Palatability chocolate 6.39 (2.53)^1^ 7.24 (2.02) 7.29 (2.36) *F*=2.26, *p*=.11

Awareness of signal-stimuli 12.8 6 ^1^ 6.5 ^2^ *χ^2^*=1.75, *p*=.42

association (% of participants)

Task increased intake 21 12 ^1^ 13 ^2^ *χ^2^*=1.89, *p*=.39

(% of participants)

^1^Data missing from one participant in this group

^2^Data missing from two participants in this group

**Supplementary Table 4. Details of exclusions in each experiment.** This table shows the number of participants excluded from each experimental condition following screening for the identified exclusion criteria.

Experiment 1 1 2 2 2 3 3 3

Group Stop Double Stop Double Ignore Stop Double General Stop

Reason for exclusion

Previous participation in

a similar study 3 4 5 4 3 2 1 2

Eaten < 3 hours ago 0 1 1 0 2 0 4 1

Unable to complete taste test^1^ 0 0 2 2 0 1 2 1

Incomplete response files^2^ 0 0 3 1 0 1 1 0

Intake > 3 SD group mean 0 0 0 1 1 1 0 0

Go accuracy < 3 SD group mean 1 1 1 2 0 1 1 1

Signal accuracy < 3 SD^3^ group mean 0 1 3 1 2 2 0 2

Total exclusions from each group **4 7 15 11 8 8 9 7**

Total exclusions from experiment **11** **34** **24**

^1^Participants were unable to complete the taste test due to fasting (n = 7) or leaving the study early (n = 1)

^2^Technical faults resulted in some incomplete response files so performance could not be verified

^3^An exclusion threshold of signal trial performance lower than 3 SDs from the group mean was applied to participants in the double or ignore groups. A more conservative threshold, lower than 2 SDs from the group mean, was applied to participants in the inhibition (stop) groups because 3 SDs included 0% stop-signal accuracy in some studies, and we only wanted to retain participants who showed some evidence of successful stopping.
